# Molar Incisor Hypomineralization: A Survey of Dental Students from Polish Medical Universities

**DOI:** 10.3390/pediatric16040086

**Published:** 2024-11-18

**Authors:** Wojciech Tynior, Daria Pietraszewska, Magdalena Truszkowska, Danuta Ilczuk-Rypuła

**Affiliations:** 1 Department of Medical and Molecular Biology, Faculty of Medical Sciences in Zabrze, Medical University of Silesia in Katowice, 19 Jordana St., 41-808 Zabrze, Poland; 2 Department of Pediatric Dentistry, Faculty of Medical Sciences in Zabrze, Medical University of Silesia in Katowice, 2 Traugutta Sq., 41-800 Zabrze, Poland

**Keywords:** MIH, dental students, awareness, molar incisor hypomineralization, Poland

## Abstract

Background: Molar incisor hypomineralization (MIH) is a congenital qualitative disorder of the enamel tissue. During examinations, clinicians may observe a range of enamel opacities during examinations. The aim of this study was to assess the knowledge and awareness of dental students in Poland regarding MIH. Methods: This study was conducted among dental students who participated in scientific conferences for dental students in Poland. An electronic questionnaire was created for this purpose, accessible via a QR code. Results: The respondents consisted of dental students from the first to the fifth year of study, including 98 fourth-year students and 76 fifth-year students. The students represented 10 medical universities in Poland. Of the 341 respondents, 256 (75.07%) stated that they were familiar with the term MIH, but only 61 (17.89%) correctly indicated the diagnostic criteria of MIH. The most frequently indicated condition resembling MIH was enamel hypoplasia, with 158 (46.33%) respondents identifying it. Conclusions: Dental students at Polish medical universities have basic knowledge of MIH, but it is insufficient and needs to be improved during their educational training.

## 1. Introduction

Molar incisor hypomineralization (MIH) is a congenital qualitative disturbance of enamel tissue [1,2]. The worldwide prevalence of MIH ranges from 0.5% to 40.2% according to different studies [3]. In 2003, the European Academy of Pediatric Dentistry presented judgement criteria for MIH [1,2]. Diagnosis is confirmed when at least one permanent first molar is affected. Permanent incisors might also be involved. The clinical manifestations of MIH vary diversely to the severity of the disease, with opacities ranging from white to creamy to yellow and brown. In severe cases, post-eruptive enamel breakdown, atypical restorations, extraction of molars, or failure of molars or incisors to erupt may be observed [2,4,5].

The diagnostic criteria of MIH have been established for over 20 years, but diagnosis can be challenging due to similar dental defects, complex clinical manifestations, and previously performed treatment. A major complication is the wide variety of clinical views of the disease and the increasing prevalence of MIH. Delayed diagnosis can lead to extensive dental damage and the necessitate complex treatment. The problem of MIH is related not only to proper diagnosis and effective treatment, but also to the psychological wellbeing of the child and his/her parents [6,7,8,9]. Changes in the aesthetic zone can cause patients fear, shame, and anxiety. Children with MIH may also experience stigma or bullying from peers at school.

To minimise the adverse effects of MIH, such as complex clinical presentations and psychological challenges, it is essential to increase dentists’ awareness of this emerging dental issue. This could enable a faster and more precise diagnosis, minimising the disease’s impact on children’s quality of life.

Dental education in Poland is structured over five years. The first two years focus on basic science, providing students with a foundation knowledge base. This is followed by pre-clinical training, which effectively bridges the gap between theoretical knowledge and practical clinical skills. From the third year, students begin clinical training, which allows them to apply their learning in a clinical setting with patients. Assessing awareness among dental students is one of the most accessible ways to gauge preparedness among future dental professionals. Through proper academic education, students acquire knowledge that can be applied in clinical practice from their first day of work.

The aim of this study was to assess knowledge and awareness of MIH among Polish dental students and to evaluate the need for greater emphasis on MIH in academic education.

## 2. Materials and Methods

### 2.1. Study Design

An electronic questionnaire was prepared to assess dental students’ knowledge and awareness of MIH. The survey was based on previously conducted studies in Austria and Germany [10,11].

The first section collected the respondents’ personal data (age, gender, year of study, name of university, verification of student status). The second section questions the knowledge and definition of MIH, the diagnostic criteria, sources of knowledge, aetiology, and certainty regarding MIH diagnosis. The final section addressed the level of education and the need to expand on certain topics related to MIH in university courses. Three authors validated the questions and usability of the electronic form before launching the questionnaire. No similar research has been conducted in Poland.

### 2.2. Setting Participants and Data Colletion

This study was conducted among dental students attending three scientific conferences organised by the Polish Association of Dental Students (PTSS) in Katowice, Wroclaw, and Lublin. The conferences were addressed to all dental students and early-career dentists. Data collection took place from January 2022 to June 2023.

Conference participants received access to the surveys via a QR code that directed them to the online version of questionnaire. Only dental students who correctly completed the survey on their personal electronic device (phone/tablet) were included. Two separate questions were used to verify the respondents’ student status. Thirteen questionnaires were rejected because they were completed by graduate-dentists in their first year of practice. All records were transferred to an Excel spreadsheet.

### 2.3. Statistical Analysis

Only fully completed questionnaires were analysed. Statistical analysis was performed using Excel (Microsoft) and Statistica (TIBCO Software Inc), version 13.

## 3. Results

A total of 354 questionnaires were received, of which 13 were excluded because they did not meet the inclusion criteria. The respondents of the survey were Polish dental students, ranging from first year to fifth year and representing 10 different Polish medical universities. The number of respondents was as follows: 33 first-year, 55 second-year, 79 third-year, 98 fourth-year, and 76 fifth-year students.

In Poland, the first two years and part of the third year of dental studies are devoted to basic sciences, with clinical training beginning in the third year. Subsequently, in the fourth and fifth years, the topic of MIH is introduced and discussed.

Our primary focus was on students in the last two years of their dental education. The results were categorised into four groups: first to fifth year, first to third year, fourth year, and fifth year.

Of the 341 respondents, 256 (75.07%) stated that they were familiar with the term MIH, including 91 (92.86%) in their fourth year and 76 (100%) in their fifth year.

The most frequently cited sources of knowledge were academic lectures and discussions (222 (86.72%)), academic books (220 (85.94%)), and external lectures and conferences (134 (52.34%)).

Among the 256 respondents familiar with the term MIH, 108 (42.19%) claimed to have knowledge of the diagnostic criteria for MIH, including 37 (40.66%) fourth-year students and 60 (78.95%) fifth-year students. However, only 61 (23.83%) correctly indicated the criteria, including 20 (21.98%) fourth-year students and 41 (53.95%) fifth-year students.

The certainty of the MIH diagnosis was also assessed. For the total group, the mean was 2.93 on a scale of 1–5, including 2.71 for fourth-year students and 3.09 for fifth-year students.

The respondents were also asked about dental conditions similar to MIHA; a total of 176 (51.61%) people indicated knowledge of such conditions, with the most frequently mentioned being enamel hypoplasia (158 (61.72%)), amelogenesis imperfecta (145 (56.64%), and fluorosis (123 (48.05%)). Table 1 presents more detailed data.

Concerning the aetiology of MIH, 169 (66.02%) students described it as “not being clearly defined”, including 62 (81.58%) fourth-year students and 67 (73.63%) fifth-year students. The most common aetiology factors for the total group that were indicated were genetic factors, 176 (68.75%), drugs in pregnancy (169 (66.02%), and prematurity, 163 (63.67%). However, in the group of fifth-year students, the most common responses were infection at up to 3 years of age (67 (88.16%)), high fever at up to 3 years of age (64 (84.21%)), and prematurity (64 (84.21%)). Table 2 provides further details.

The authors asked the respondents to identify three of the most important problems regarding the treatment of a patient with MIH. The answers were as follows: young age of the patient—161 (62.89%) respondents, quick caries development—133 (51.95%) respondents, and difficulties in maintaining dental fillings—118 (46.09%) respondents. Similar trends were observed among the fifth-year students who also highlighted young patient age (49 (64.47%)), the difficulty in maintaining dental fillings (44 (57.89)), and the quick progression of caries (44 (57.89%)) as significant issues. Table 3 provides additional data regarding MIH.

The last section of the questionnaire investigated the necessity for enhanced academic education in the field of MIH. Students indicated the importance of greater education for treatment, 225 (65.98%), diagnostics, 224 (65.69%), and aetiology, 164 (48.09%), for MIH. Similar answers were provided by fifth-year students: treatment (45 (59.21%)), diagnostics (39 (51.32%), and aetiology (20 (26.32%)). Table 4 provides further details.

## 4. Discussion

Our study analysed the awareness and knowledge of Polish dental students regarding MIH. The available Polish papers have focused on different aspects of MIH. Previous studies have investigated the prevalence, severity, dentist awareness, and treatment options of patients with MIH [12,13,14,15,16,17]. This study is the first that assess the awareness and knowledge of MIH among dental students in Poland.

Research on dental students has been conducted in Germany, Switzerland, Austria, Turkey, Egypt, Saudi Arabia, Syria, and China [10,11,18,19,20,21,22,23]. The studies were conducted from 2016 to 2023. The surveys investigated students in their final year of study. The size of the groups varied across countries, with the largest number of participants in Syria (867 participants) [18]. The authors used paper or digital forms of the questionnaire. They included questions that were similar to previous studies, available in public medical databases.

In our survey, all of the final-year students (100%) and 91 of the fourth-year students (92.86%) were familiar with the term MIH. The results align closely with findings reported by other researchers [11,19,20,22,24]. Notably, the Saudi Arabian survey indicated that a significant majority of participants (64%) were not aware of the term “MIH” [21].

In our survey, we also asked about the main source of knowledge about MIH. Polish students indicated that lectures and discussions during academic education (222 (86.72%)), academic books (220 (85.94%)), and external lectures and conferences (134 (52.34%)) were the most frequent sources of knowledge. Other authors prepared this question in a different style. However, they present the same general result, that the most influential sources were different forms of university education, such as academic books, academic discussions, and lectures [11,19,20,21,22,24]. The presented trend highlights the importance of academic institutions in providing comprehensive education.

Our study also assessed students’ confidence in diagnosing MIH. The mean confidence score for fifth-year students was 3.09. Similar questions were included in the other authors’ questionnaires. However, it is difficult to undertake a comparison due to the different methodologies. Most authors used a four-degree descriptive scale (“Very confident”, “Confident”, “Slightly confident”, “Not confident at all”). Among the three surveys in Egypt, Switzerland, and Turkey, the most frequent response was “Slightly confident”. This was followed by the answer “Not confident” in the Egyptian and Swiss student groups and the answer “Confident” in Turkey [19,20,23].

Polish fifth-year students selected enamel hypoplasia 63 (82.89%), amelogenesis imperfecta 63 (82.89%), and fluorosis 48 (63.16%) as the most frequently reported dental conditions that may be similar to MIH. Other authors formulated this question differently. They asked the students about conditions that might create difficulties in distinguishing between MIH and other conditions. Enamel hypomineralization, amelogenesis imperfecta, and fluorosis were indicated as the most common answers [10,11,18,19,20,23].

According to our study, Polish final-year students have suggested that the most common cause of MIH is childhood infection up to the age of 3 (67 (88.16%)). By contrast, genetic factors were most frequently cited in studies form Syria, Egypt, Switzerland, Turkey, Germany, and Austria [10,11,18,19,20,21]. In the group of Polish students, a genetic factor also had a high score of 58 (76.32%). However, it was ranked 10th after other factors, such as infection up to the age of 3, prematurity, a high fever up to the age of 3, low birth weight, smallpox, viral infections during pregnancy, measles, bronchitis, and pneumonia.

The most common clinical challenge for Polish students was “young age of the patient”. However, students from many countries reported the problem of the long-term maintenance of dental fillings as the most common clinical problem [10,11,18,19]. In our survey, this term was described differently as “difficulty in maintaining the dental filling”, and it came in second place.

Polish students emphasised the need for further education in the treatment (225 (65.98%)), diagnosis (224 (65.69%)), and aetiology (164 (48.09%)) of MIH. Similar observations have been made in studies conducted in other countries [10,11,18,19]. In the results of several studies, treatment and diagnosis appear in the first two places.

This study was limited by the insufficient number of students per year and the variability across different universities. Therefore, it cannot be considered population-based research. To overcome these limitations and improve the accuracy of this study, we recommend conducting a larger-scale multicentre study.

## 5. Conclusions

Dental students at Polish medical universities have a basic knowledge of MIH. We observed an increasing trend in the field of knowledge within the years of study. They express a desire to develop their knowledge in this area during their academic education. Despite this interest, their knowledge is insufficient and needs to be improved.

## Figures and Tables

**Table 1:** The knowledge and perception among Polish dental students of MIH.

Question	All Students		First–Third Year		Fourth Year		Fifth Year	
	(n = 341)		(n = 167)		(n = 98)		(n = 76)	
Are you familiar with the term “MIH”?								
Yes	256	75.07%	89	53.29%	91	92.86%	76	100.00%
Where do you obtain your knowledge about MIH?	The following questions were addressed to those students who indicated that they were familiar with the term “MIH”.							
	n = 256		n = 89		n = 91		n = 76	
External lectures and conferences	134	52.34%	54	60.67%	44	48.35%	36	47.37%
Academic books	220	85.94%	69	77.53%	78	85.71%	73	96.05%
Articles and medical databases	131	51.17%	49	55.06%	43	47.25%	39	51.32%
Social media	133	51.95%	47	52.81%	44	48.35%	42	55.26%
Personal experience (MIH among family members)	66	25.78%	27	30.34%	18	19.78%	21	27.63%
Lectures and discussions during university education	222	86.72%	72	80.90%	75	82.42%	75	98.68%
Other students	112	43.75%	46	51.69%	31	34.07%	35	46.05%
Dental professionals	108	42.19%	43	48.31%	38	41.76%	27	35.53%
Video platforms	78	30.47%	31	34.83%	26	28.57%	21	27.63%
Websites for patients	53	20.70%	21	23.60%	17	18.68%	15	19.74%
Other	21	8.20%	9	10.11%	7	7.69%	5	6.58%
Do you know the criteria for MIH diagnosis?								
Yes	108	42.19%	11	12.36%	37	40.66%	60	78.95%
What is the necessary symptom to diagnose MIH?								
At least one permanent molar affected	61	23.83%	0	0.00%	20	21.98%	41	53.95%
On a scale of 1-5, how confident are you in making the diagnosis of MIH?								
	Mean = 2.93		Mean = 2.00		Mean = 2.71		Mean = 3.09	
Do you know of any other enamel defects that may be similar to MIH?								
Yes	176	69.29%	34	38.20%	74	81.32%	68	89.47%
	The following questions were addressed to those students who indicated that they know of diseases and dental defects similar to MIH.							
	n = 176		n = 34		n = 74		n = 68	
Amelogenesis imperfecta	145	56.64%	23	25.84%	59	64.84%	63	82.89%
Enamel hypoplasia	158	61.72%	15	16.85%	67	73.63%	63	82.89%
White spotted lesion	107	41.80%	57	64.04%	42	46.15%	46	60.53%
Tooth abrasion	43	16.80%	107	120.22%	15	16.48%	23	30.26%
Caries lesion	69	26.95%	80	89.89%	31	34.07%	34	44.74%
Dentinogenesis imperfecta	80	31.25%	75	84.27%	35	38.46%	35	46.05%
Dental erosion	76	29.69%	79	88.76%	36	39.56%	30	39.47%
Fluorosis	123	48.05%	45	50.56%	52	57.14%	48	63.16%
Enamel attrition	39	15.23%	108	121.35%	13	14.29%	24	31.58%
Enamel abfraction	25	9.77%	122	137.08%	10	10.99%	13	17.11%

**Table 2:** The knowledge of MIH aetiology among Polish dental students.

Question	All Students		First–Third Year		Fourth Year		Fifth Year	
	(n = 256)		(n = 89)		(n = 91)		(n = 76)	
Is the aetiology of MIH clearly defined?	Question for those students who are familiar with MIH.							
Yes	17	6.64%	6	6.74%	5	5.49%	6	7.89%
No	169	66.02%	40	44.94%	67	73.63%	62	81.58%
Don’t know	70	27.34%	43	48.31%	19	20.88%	8	10.53%
Viral mother infections during pregnancy	158	61.72%	40	44.94%	59	64.84%	59	77.63%
Drugs used in pregnancy	169	66.02%	48	53.93%	65	71.43%	56	73.68%
Prematurity	163	63.67%	38	42.70%	61	67.03%	64	84.21%
Low birth mass	150	58.59%	34	38.20%	56	61.54%	60	78.95%
Labour complications	125	48.83%	24	26.97%	45	49.45%	56	73.68%
Environmental pollutants	114	44.53%	29	32.58%	39	42.86%	46	60.53%
Infection at up to 3 years	154	60.16%	39	43.82%	48	52.75%	67	88.16%
High fever at up to 3 years	128	50.00%	24	26.97%	40	43.96%	64	84.21%
Smallpox	120	46.88%	24	26.97%	36	39.56%	60	78.95%
Measles	118	46.09%	22	24.72%	38	41.76%	58	76.32%
Bronchitis	112	43.75%	21	23.60%	33	36.26%	58	76.32%
Asthma	103	40.23%	22	24.72%	30	32.97%	51	67.11%
Otitis	104	40.63%	19	21.35%	29	31.87%	56	73.68%
Pneumonia	107	41.80%	16	17.98%	33	36.26%	58	76.32%
Genetic factors	176	68.75%	52	58.43%	66	72.53%	58	76.32%
Dioxins	102	39.84%	29	32.58%	35	38.46%	38	50.00%
Bisphenol A	103	40.23%	28	31.46%	40	43.96%	35	46.05%
Vaccinations	14	5.47%	2	2.25%	2	2.20%	10	13.16%
COVID vaccinations	10	3.91%	1	1.12%	3	3.30%	6	7.89%
Fluor intake	42	16.41%	12	13.48%	16	17.58%	14	18.42%
Food allergy	45	17.58%	8	8.99%	9	9.89%	28	36.84%

**Table 3:** The perception of clinical challenges among Polish dental students.

Question	All Students		First–Third Year		Fourth Year		Fifth Year	
	(n = 256)		(n = 89)		(n = 91)		(n = 76)	
Indicate three the most important problems regarding the treatment of a patient with MIH	Question for those students, who are familiar with MIH.							
I don’t know	40	15.63%	30	33.71%	9	9.89%	1	1.32%
Young age of the patient	161	62.89%	46	51.69%	66	72.53%	49	64.47%
Lack of acceptance among friends	47	18.36%	17	19.10%	19	20.88%	11	14.47%
Fast development of pulpitis	73	28.52%	18	20.22%	22	24.18%	33	43.42%
Quick caries development	133	51.95%	38	42.70%	51	56.04%	44	57.89%
Teeth hypersensitivity	81	31.64%	20	22.47%	27	29.67%	34	44.74%
Lack of full dental anaesthesia	1	0.39%	1	1.12%	0	0.00%	0	0.00%
Difficulties in maintaining dental filling	118	46.09%	25	28.09%	47	51.65%	46	60.53%

**Table 4:** Educational needs in MIH among Polish dental students.

Question	All Students		First-Third Year		Fourth Year		Fifth Year	
	(n = 341)		(n = 167)		(n = 98)		(n = 76)	
What topics should be expanded during study?								
Aetiology	164	48.09%	88	52.69%	56	57.14%	20	26.32%
Diagnostics	224	65.69%	113	67.66%	72	73.47%	39	51.32%
Treatment	225	65.98%	111	66.47%	69	70.41%	45	59.21%
Psychological aspect	120	35.19%	63	37.72%	41	41.84%	16	21.05%

## Data Availability

The raw data supporting the conclusions of this article will be made available by the authors on request.
